# Anomaly Detection in Host Signaling Pathways for the Early Prognosis of Acute Infection

**DOI:** 10.1371/journal.pone.0160919

**Published:** 2016-08-17

**Authors:** Kun Wang, Stanley Langevin, Corey S. O’Hern, Mark D. Shattuck, Serenity Ogle, Adriana Forero, Juliet Morrison, Richard Slayden, Michael G. Katze, Michael Kirby

**Affiliations:** 1 Department of Mathematics, Colorado State University, Fort Collins, CO, United States of America; 2 Department of Mechanical Engineering & Materials Science, Yale University, New Haven, CT, United States of America; 3 Department of Microbiology, School of Medicine, University of Washington, Seattle, WA, United States of America; 4 Department of Applied Physics, Department of Physics, and Graduate Program in Computational Biology & Bioinformatics, Yale University, New Haven, CT, United States of America; 5 Department of Physics and Benjamin Levich Institute, The City College of the City University of New York, New York, NY, United States of America; 6 Department of Microbiology, Immunology and Pathology, Colorado State University, Fort Collins, CO, United States of America; 7 Department of Biomedical Sciences, Colorado State University, Fort Collins, CO, United States of America; 8 Department of Computer Science, Colorado State University, Fort Collins, CO, United States of America; University of Pittsburgh, UNITED STATES

## Abstract

Clinical diagnosis of acute infectious diseases during the early stages of infection is critical to administering the appropriate treatment to improve the disease outcome. We present a data driven analysis of the human cellular response to respiratory viruses including influenza, respiratory syncytia virus, and human rhinovirus, and compared this with the response to the bacterial endotoxin, Lipopolysaccharides (LPS). Using an anomaly detection framework we identified pathways that clearly distinguish between asymptomatic and symptomatic patients infected with the four different respiratory viruses and that accurately diagnosed patients exposed to a bacterial infection. Connectivity pathway analysis comparing the viral and bacterial diagnostic signatures identified host cellular pathways that were unique to patients exposed to LPS endotoxin indicating this type of analysis could be used to identify host biomarkers that can differentiate clinical etiologies of acute infection. We applied the Multivariate State Estimation Technique (MSET) on two human influenza (H1N1 and H3N2) gene expression data sets to define host networks perturbed in the asymptomatic phase of infection. Our analysis identified pathways in the respiratory virus diagnostic signature as prognostic biomarkers that triggered prior to clinical presentation of acute symptoms. These early warning pathways correctly predicted that almost half of the subjects would become symptomatic in less than forty hours post-infection and that three of the 18 subjects would become symptomatic after only 8 hours. These results provide a proof-of-concept for utility of anomaly detection algorithms to classify host pathway signatures that can identify presymptomatic signatures of acute diseases and differentiate between etiologies of infection. On a global scale, acute respiratory infections cause a significant proportion of human co-morbidities and account for 4.25 million deaths annually. The development of clinical diagnostic tools to distinguish between acute viral and bacterial respiratory infections is critical to improve patient care and limit the overuse of antibiotics in the medical community. The identification of prognostic respiratory virus biomarkers provides an early warning system that is capable of predicting which subjects will become symptomatic to expand our medical diagnostic capabilities and treatment options for acute infectious diseases. The host response to acute infection may be viewed as a deterministic signaling network responsible for maintaining the health of the host organism. We identify pathway signatures that reflect the very earliest perturbations in the host response to acute infection. These pathways provide a monitor the health state of the host using anomaly detection to quantify and predict health outcomes to pathogens.

## Introduction

Upon infection, human pathogens (bacteria, fungi, parasites, and viruses) induce a complex cascade of host responses that have evolved to detect the pathogen and minimize the disease severity [1]. This multicellular signaling network is triggered by pathogen-specific motifs and intracellular perturbations that activate/recruit host immune cells to infected sites and induce cell death of infected cells. The host’s ability to sense and control pathogen replication is primarily accomplished by the immune system. Both the innate and adaptive immune response to a particular infectious agent is deliberate and dictated by a carefully orchestrated sequence of host signaling networks. By characterizing the pathogen-specific host signaling networks and the timing at which the pathways activate following infection, host-derived clinical assays could be developed to augment current medical diagnostic capabilities for acute infectious diseases.

Early diagnosis of an acute infection is critical to quickly select the appropriate medical intervention for optimum patient care and improve the overall disease outcome. While most clinical diagnostic assays rely on pathogen detection, advances in technologies (e.g. sequencing, microarrays, mass spec.) to measure the host response to infection provide a wealth of data that can be exploited to improve infectious disease diagnostics [2]. Despite the efforts of many groups for over a century, the search for host-derived biomarkers indicative of infection has remained elusive. Recent studies have successfully applied host gene expression and proteomics data sets to identify host conical pathways and/or individual genes associated with a particular infectious disease [3–5]. Algorithms from machine learning have been increasingly used to identify discriminative genes to characterize an organism’s biological state, see, e.g., [6–12]. However, the challenge in such studies is to bridge the gap between single genes that serve a discriminative function from those that provide insight into the biological process of disease.

In order to enhance information that may be obtained by the analysis of single genes, pathway-based analysis has become increasingly popular as an approach to elucidate the underlying biological processes under investigation [13, 14]. Instead of focusing on selection of single discriminative host genes associated with infection, pathways are a collection of predefined sets of genes that are known to be involved in a particular cellular or physiologic function. By quantifying the gene expression levels within a particular pathway, the pathway-based methods select and rank the pathways most associated with the disease state to improve the accuracy of the host genes defined by the computational analysis and the biological interpretation of the results. Several pathway analytics have been developed to identify host-signaling networks for biological states classification and prognosis. For example, in one influenza study, the top 100 discriminatory genes can be removed from the analysis without a drop in classification accuracy motivating a pathway analysis involving the top 1500 genes [15].

In this study we investigate the human cellular response to infection by treating the changes in gene expression as a problem in anomaly detection. Healthy individuals are assumed to be in a homeostatic state with immune systems that are expressing nominally while individuals who are becoming sick possess a cascade of pathways that reflect the systematic response to a specific invading pathogen. Our goal was to elucidate pathway-based signatures that may aid in diagnosis as well as early prognosis of acute infection. We employed a pathway-based implementation of the Multivariate State Estimation Technique (MSET), a method for detecting anomalies where the nominal data possesses substantial nonlinear structure in temporally evolving systems [16–21]. This approach allowed us to analyze temporal data sets by detecting host gene regulation anomalies within functional host networks during transitions between biological states (i.e., healthy to symptomatic). Host pathways induced by exposure to a particular infectious agent are ranked based on predictive accuracy and then pathways that highly correlate with the disease state are ranked according to when they deviate from a healthy baseline state. By incorporating the temporal dimension in our pathway analysis model, we have constructed an approach that identifies early host signaling pathways or clinical biomarkers for diagnosing acute infectious diseases and for predicting the disease outcome.

We evaluated the anomaly detection approach using temporal gene expression data sets to identify early host functional pathways associated with acute respiratory infections in humans. Acute respiratory disease is a common diagnosis in clinical settings and a major cause of mortality worldwide [22]. The high prevalence of bacteria and virus species that contribute to the global respiratory disease burden combined with a significant rate of respiratory related co-infections make the development of diagnostic tools particularly challenging. Respiratory viruses such as influenza virus, respiratory syncytia virus, and human rhinovirus, are significant public health threats and represent the majority of respiratory infections reported in clinical settings. The rampant overuse of antibiotics in clinics to treat acute respiratory infections has led to the emergence of antibiotic-resistant bacteria strains limiting our medical interventions for pathogenic bacterial infections. The identification of host biomarkers to distinguish between bacterial and viral respiratory infections is critical to changing this current paradigm. We applied MSET to define host pathways associated with acute respiratory virus infection and ranked pathways temporally to identify early host biomarkers that predict infection status and disease outcome.

## Results

In order to test and validate the methodology, we explore MSET for biological early warning using data generated by a mathematical model of the immune system’s response to infection as well as gene expression data sets arising in influenza and endotoxin experiments.

There are only a limited number of gene expression data sets that measure the human immune system’s response to infection. Generally they have low temporal resolution, e.g., samples every 8 hours, but detailed gene coverage that allows us to perform a pathway based modeling approach. Real data sets also typically have a small number of subjects [4, 5, 23]. In contrast, the numerical simulations of virtual patients can generate finely sampled data in time for potentially millions of subjects but, at least for the example we consider, capture only a limited number of variables. Thus the real and numerical datasets each have aspects that provide different challenges to the algorithm.

In what follows, we first use the numerical simulation data to test MSET’s effectiveness for the detection and prognosis of sepsis. We then proceed with a more realistic proof of concept concerning the ability to use pathway analysis coupled with failure prediction algorithms for early warning of a biological disease.

### Early Warning on a Mathematical Model

To illustrate the performance of the early warning algorithm on a large dataset, we use a mathematical model to simulate the immune system’s response to infection. The model [24] describes the immune response in terms of the levels of pathogen *P*, activated phagocytes (neutrophils) *N*, tissue damage *D*, and anti-inflammatory mediators *C*_*A*_. This model consists of four ordinary differential equations:

$$\begin{array}{cc} \frac{dP}{dt} & =k_{pg}P\left(1-\frac{P}{p_{\infty }}\right)-\frac{k_{pm}s_{m}P}{\mu_{m}+k_{mp}P}-k_{pn}f\left(N\right)P,\\ \frac{dN}{dt} & =\frac{s_{nr}R}{\mu_{nr}+R}-\mu_{n}N,\\ \frac{dD}{dt} & =k_{dn}\frac{f{\left(N\right)}^{6}}{x_{dn}^{6}+f{\left(N\right)}^{6}}-\mu_{d}D,\\ \frac{dC_{A}}{dt} & =s_{c}+k_{cn}\frac{f(N+k_{cnd}D)}{1+f(N+k_{cnd}D)}-\mu_{c}C_{A}, \end{array}$$

where *R* = *f*(*k*_*nn*_*N* + *k*_*np*_*P* + *k*_*nd*_*D*), and $f\left(x\right)=\frac{x}{1+\frac{C_{A}}{c_{\infty }}}$.

The system characterizes the time evolution of these four variables, which we view metaphorically as proxies for gene expression. The model admits three final states under certain parameter choices (see [24] for details): 1) Healthy (*H*): (*P*, *N*, *D*, *C*_*A*_) = (0, 0, 0, *C*_*A*_) for *C*_*A*_ > 0, 2) Aseptic (*S*_0_): (*P*, *N*, *D*, *C*_*A*_) = (0, *N*, *D*, *C*_*A*_) for *N*, *D*, *C*_*A*_ > 0, and 3) Septic (*S*_1_): (*P*, *N*, *D*, *C*_*A*_) = (*P*, *N*, *D*, *C*_*A*_) for all components positive.

A virtual time course experiment is performed by varying the reference parameters and the initial conditions for *P* and *C*_*A*_ in the above equations to reflect virtual subject’s variability (see [25] for details). Here 1,000 virtual patients were generated with individualized parameter profiles which were selected to simulate the three disease outcomes following the parameters proposed in [25]. The proxy expression levels were measured every hour for 168 hours for each subject. Each subject was labeled based on the final state. The distribution of final states is: *H* = 597, *S*_0_ = 224, and *S*_1_ = 176. Three subjects that did not reach steady-state criteria were excluded.

**Fig 1:**
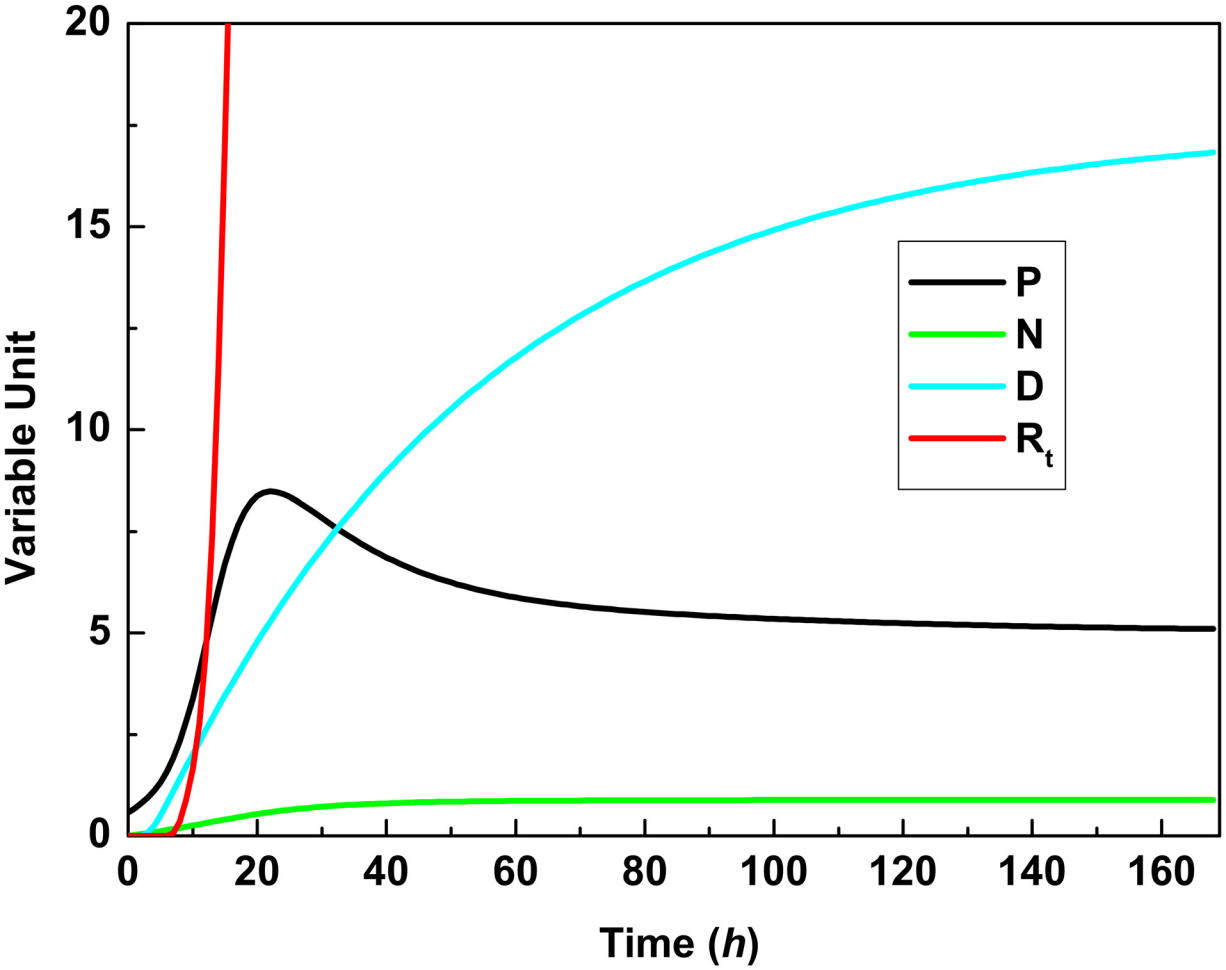
Evaluation of MSET to detect host pathway anomalies. An example of a subject whose model goes into alarm (the residuals *R*_*t*_ are indicated by the red line) providing an early indication of a symptomatic outcome. The simulated disease evolves under the governing system of differential equations and the model goes into alarm as the pathogen (P) starts to increase substantially in concentration. The neutrophils (N) and the damage (D) also start to grow as the system becomes anomalous.

For MSET analysis, the healthy data set denoted by *H* is equally divided into two parts randomly to create the memory matrix *D* from training 299 points, and the test data set *T*_0_ of size 298. (Given the largely different contexts in which they occur, the reader should not confuse the data matrix *D* associated with MSET and the scalar variable *D* representing damage in the dynamical systems model.) The symptomatic data sets *S*_0_ and *S*_1_ serve as test data to evaluate the model. An example simulation of a patient is shown in Fig 1. We see that the residual error *R*_*t*_ in the MSET model grows quickly indicating early that this subject is becoming symptomatic. We observe this residual growing before 10 hours have elapsed indicating that the patient will become septic. After 20 hours the pathogen is brought under control by the immune response but the damage continues to increase. We emphasize that this model does not incorporate a therapy that would presumably be administered after the early prognosis. Table 1 shows that MSET can effectively predict disease outcome averaging 7.8 hours for *S*_0_ and 6.3 hours for *S*_1_, the most severe outcome, i.e., septic death.

**Table 1:** MSET performance on synthetic data. Accuracy (Acc) is the percentage of subjects who are correctly identified as having the actual states. Prognosis Time (Time) is calculated based on correctly identified subjects. Only true positive (actual disease) subjects can have meaningful prognosis time.

Data	Acc(%)	Time (*h*)
*T*_0_	99.3	
*S*_0_	93.3	7.8
*S*_1_	100	6.3

**Fig 2:**
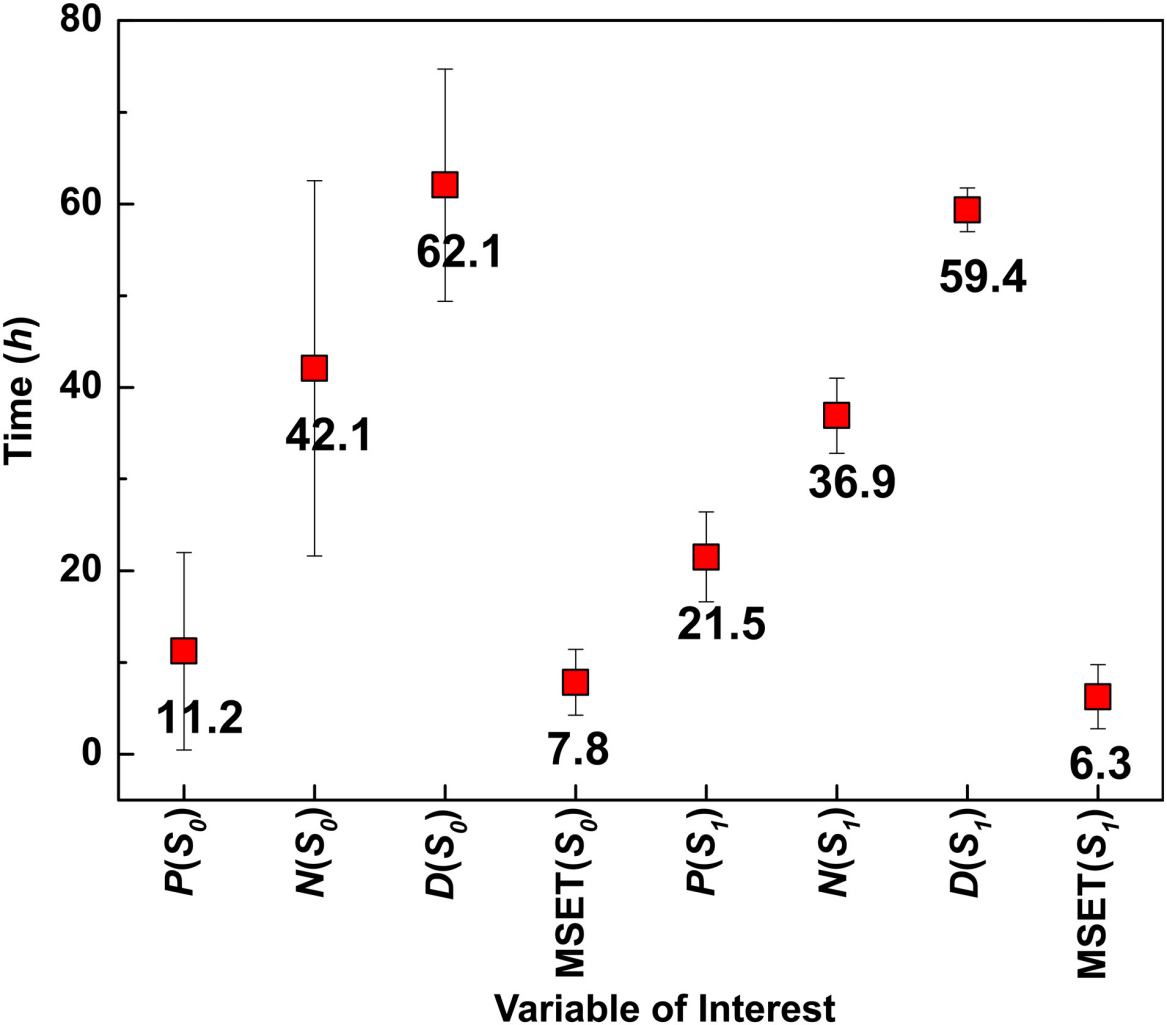
Statistical parameters for MSET analysis. The statistical measures of prognosis time (MSET) and peak expression time in the simulated model variables pathogen (P), neutrophils (N) and the damage (D) for both the asceptic (*S*_0_) and septic death (*S*_1_) parameters. MSET anomaly detection times are substantially earlier than variable peak expressions.

The statistical measures of prognosis time, mean and standard deviation are shown in Fig 2 for both *S*_0_ and *S*_1_. The time of peak expression levels for each variable is shown for purposes of comparison. It is interesting to observe that the MSET model outperforms any model based on the use of a single variable using the time to peak expression. Despite the early occurrence of damage shown in Fig 1 we see that damage is actually the slowest variable for predicting outcomes because it takes the longest to peak. MSET predicts outcomes for patients who will become symptomatic on the order of 6-8 hours after infection in this simulation. The pathogen level performs best for *S*_0_ but has high standard deviation; it’s performance degrades for *S*_1_ to over 20 hours. In summary, when compared with the model variables in Fig 2, the MSET prognosis time is consistently ahead of peak expression time. We concede that this example is only illustrative and that peak expression time is not necessarily an optimal model for early warning.

To underscore this point, we note that for this particular dynamical systems model for sepsis it is possible to numerically partition the space of initial conditions into basins of attraction that indicate the final state based solely on the initial condition without error. Hence, for this given model we can do optimal early warning trivially at *t* = 0 in the sense that the initial condition information alone contains enough information to predict final outcome. However, in general it is not possible to establish these basins of attraction for higher dimensional systems, or in the presence of noise establishing a need for alternative methods for early warning such as the one advocated here.

### Respiratory Virus Pathway Analysis

Next we apply the anomaly detection algorithm MSET to identify host cellular signaling pathways associated with the human immune response to infection by respiratory viruses. As proof of concept, we interrogated four publicly available gene expression data sets obtained from peripheral blood samples of human subjects experimentally infected with 4 different respiratory viruses, including 2 influenza virus strains: influenza viruses (H1N1, and H3N2), HRV and RSV (Table 2). In each case the model captures the nominal gene expression of the healthy state. Using these models, we identify canonical host pathways associated with the human immune response to respiratory viruses. Previous studies observed that the gene expression patterns for these four different virus were highly similar and implies there is an “acute respiratory viral” signature that is discriminative for acute respiratory infections (ARIs) [4, 5]. All subjects experimentally infected with the 4 respiratory viruses developed mild respiratory symptoms with no severe disease reported.

**Table 2:** Overview of the data sets.

Dataset	Asymp. Subjects	Sympt. Subjects	Probes	Time Points
*H*1*N*1	6	9	12023	16
*H*3*N*2	6	9	12023	16
*HRV*	10	10	12023	14
*RSV*	11	8	12023	21
*LPS*	4 (placebo)	4 (endotoxin)	22281	6

#### Classification of a Respiratory Virus Diagnostic Signature

We constructed an anomaly detection model using MSET *for each* of the 511 functional pathways used in our analysis. Each pathway model is a mapping of the identity that serves to detect any deviation from nominal, or healthy, gene expression levels for each subject whose samples are evolving in time. We expect healthy pathway expression levels will evolve into those distinctly characteristic of symptomatic and asymptomatic individuals and the analysis will classify the differentially expressed pathways in the context of host signaling networks. The analysis was performed on each dataset and ranked pathways identified in all 4 datasets were used for downstream analysis to identify robust early host signature biomarkers for respiratory virus infections.

Based on the average predictive accuracy score for each pathway, there were 16 top host pathways associated with symptomatic human subjects that met the 0.7 probability cutoff across all acute respiratory infection datasets (Table 3). Note that this accuracy measure is not directly related to early warning but we will see that these pathways also do in fact go into alarm early. These pathways represent key immune and cellular signaling networks associated with acute respiratory virus infection [26, 27]. Host pathways involved in the antiviral response (Influenza A, cytosolic DNA sensing, toll like receptor signaling, HIV/Nef), the inflammatory response (IL22BP, IL10, IL-12, African trypanosomiasis, inflammatory bowel disease, and TNFR1), and cell death/apoptosis response (Fas, lysosome, chemical) were identified as the most accurate predictors to distinguish between asymptomatic and symptomatic subjects. The top 5-ranked host pathways were IL-22BP (0.81), IL-10 (0.80), Fas (0.76), Intestinal Immune network for IgA production (0.75), and influenza A (0.74). These pathways, except for influenza A, encompass host genes expressed by pro-inflammatory immune cells (macrophages, T-cells, NK cells) and epithelial cells [28–31]. These gene networks are associated with maintaining the immune system’s homeostatic state in health and disease, primarily in the intestinal mucosa. In addition, the MSET analysis identified the KEGG influenza A pathway that contains host genes involved in the antiviral response to respiratory viruses that include early viral recognition signaling (2-5OAS/RNaseL, RIG-I, TLR7/3, and PKR) and downstream antiviral effector signaling (MxA, OAS, IFN, IL-6, TNF).

**Table 3:** Signaling Pathways selected by MSET based on the baseline (pre-inoculation) samples used as the *nominal* training data. The top pathways are selected based on the average MSET validation *T*_0_ accuracy (Acc), i.e., the percentage of subjects whose true state agree with the predicted model state. Here, only the pathways which have validation accuracy above 0.70 for all four selection in Table 5 are shown. The average MSET test *T*_1_ percentage accuracy for each pathway is also shown. The standard deviation (std) is also given. Pathways not identified as BIOCARTA are KEGG pathways.

Rank	Pathway (number of genes)	*T*_0_ Acc (std)	*T*_1_ Acc (std)
1	BIOCARTA IL22BP (14)	0.81 (0.05)	0.81 (0.06)
2	BIOCARTA IL10 (16)	0.80 (0.03)	0.77 (0.03)
3	BIOCARTA FAS (30)	0.76 (0.03)	0.76 (0.10)
4	Intestinal Immune Network IgA (42)	0.75 (0.03)	0.76 (0.08)
5	Influenza A (160)	0.74 (0.02)	0.74 (0.13)
6	African Trypanosomiasis (35)	0.74 (0.04)	0.76 (0.04)
7	Inflammatory Bowel Disease (IBD) (62)	0.74 (0.03)	0.68 (0.10)
8	Cytosolic DNA Sensing (51)	0.74 (0.02)	0.77 (0.10)
9	BIOCARTA Biopeptides (40)	0.74 (0.03)	0.75 (0.09)
10	Lysosome (114)	0.73 (0.04)	0.71 (0.10)
11	BIOCARTA Chemical (21)	0.73 (0.04)	0.70 (0.04)
12	Toll Like Receptor Signaling (99)	0.73 (0.03)	0.73 (0.07)
13	BIOCARTA IL12 (21)	0.73 (0.04)	0.73 (0.16)
14	NF-kappa B signaling (85)	0.73 (0.02)	0.72 (0.12)
15	BIOCARTA HIVNEF (56)	0.72 (0.02)	0.71 (0.08)
16	BIOCARTA TNFR1 (29)	0.71 (0.01)	0.69 (0.07)

#### Identification of Prognostic Respiratory Virus Pathway Signatures

**Fig 3:**
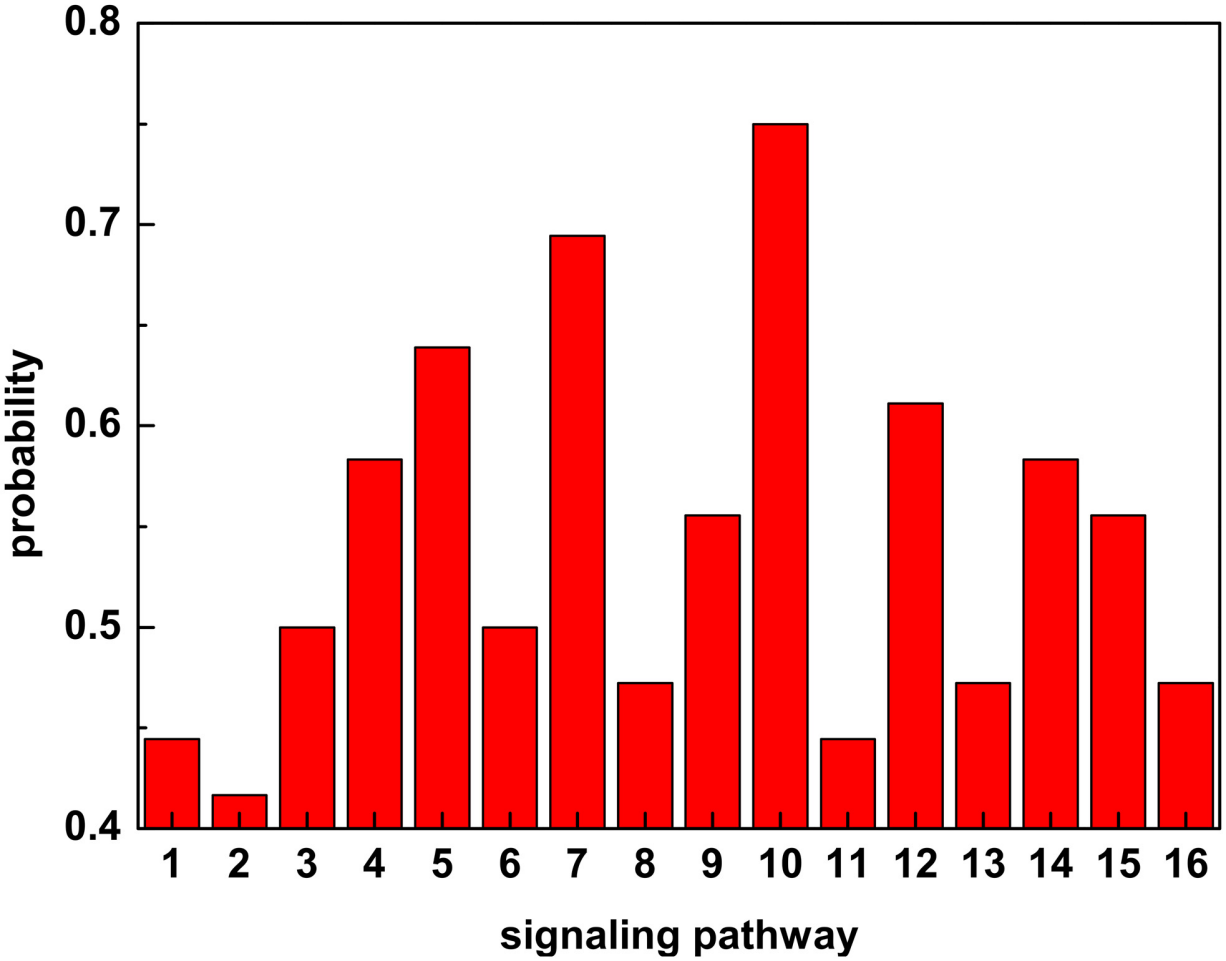
Determination of early warning pathways. The probability that each selected signaling pathway will detect the anomalous status first as computed from the test data. The signaling pathways are ranked (x-axis) based on Table 3. For example, pathway 10, KEGG Lysosome, ties or beats the other pathways over 75% of the time.

The top pathways based on diagnosis accuracy were further analyzed to identify the host signaling networks in humans predicted to deviate first from the asymptomatic or healthy state as a result of acute respiratory virus infection (Fig 3). Of central interest in this investigation are the pathways that detect anomalies on the symptomatic subjects across all four respiratory virus data sets. We determined 8 host signaling networks out of 511 pathways that alarm on at least half of the symptomatic subjects. The potential early warning pathways identified were KEGG inflammatory bowel disease, KEGG toll-like receptor signaling, KEGG Influenza A, KEGG lysosome, KEGG intestinal immune network for IgA production, BIOCARTA Biopeptides, BIOCARTA HIVNEF, and KEGG NF-kappa B signaling.

The top host signaling pathway, lysosome, is a cellular network involved with immune sensing of non-self or foreign entities in the host and is utilized by respiratory viruses to infect cells. The inflammatory bowel disease (IBD) pathway is reported in the other analyses was identified as a potential early biomarker and contains genes that influence immune system dysregulation of the mucosa, early TLR signaling, T-cell differentiation, and pro-inflammatory macrophage responses. The influenza A and TLR receptor pathways encompass gene sets that regulate host viral sensing and the antiviral response to acute respiratory virus infection in humans. The intestinal immune network for IgA production pathway plays a role in host-microbe interaction making it a natural site for the first detection of infection [31]. The top host pathways that had the lowest probability to signal first, IL22BP and IL10, are functional networks that regulate inflammatory responses and promote anti-inflammatory states. Both pathways have been shown to influence influenza virus disease severity and are associated with lung epithelial repair following influenza induced tissue damage [32, 33].

**Fig 4:**
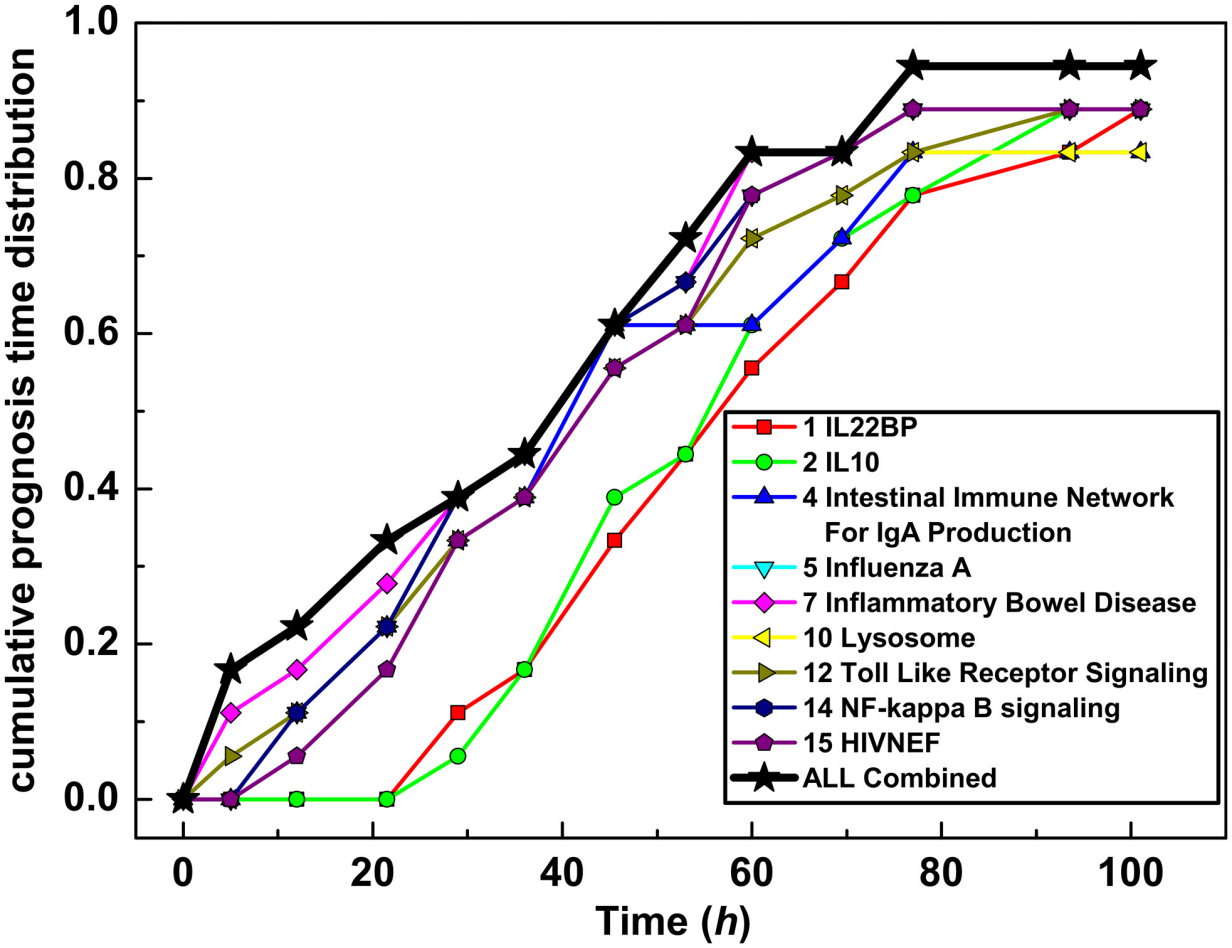
Cumulative prognosis time. The cumulative prognosis time distribution for a given pathway is the accumulated fraction of the symptomatic subjects for whom this pathway is in the alarm state as a function of time. The combined cumulative prognosis time measures the fraction of subjects who have had one or more pathways in alarm on or before the time in hours.

The performance of these pathways as early warning mechanisms for human subjects exposed to the H1N1 or the H3N2 influenza virus strain is shown in (Fig 4). The cumulative prognosis time distribution for a given pathway measures the accumulated fraction of the symptomatic subjects for whom this pathway is in the alarm state as a function of time. We note that the onset of symptoms is about the 48-60 hour range after insult while the early warning pathways suggest that almost half of the subjects will become symptomatic in less than forty hours. In fact, this is the prognosis for three subjects after only 8 hours when we use a combined criterion that triggers early warning if any of the 8 pathways are in alarm. We observe approximately 20 hours separation when comparing the two earliest pathways (lysosome and inflammatory bowel disease) with the two slowest pathways (IL-22BP and IL-10) selected from the 8 most accurate pathways.

**Fig 5:**
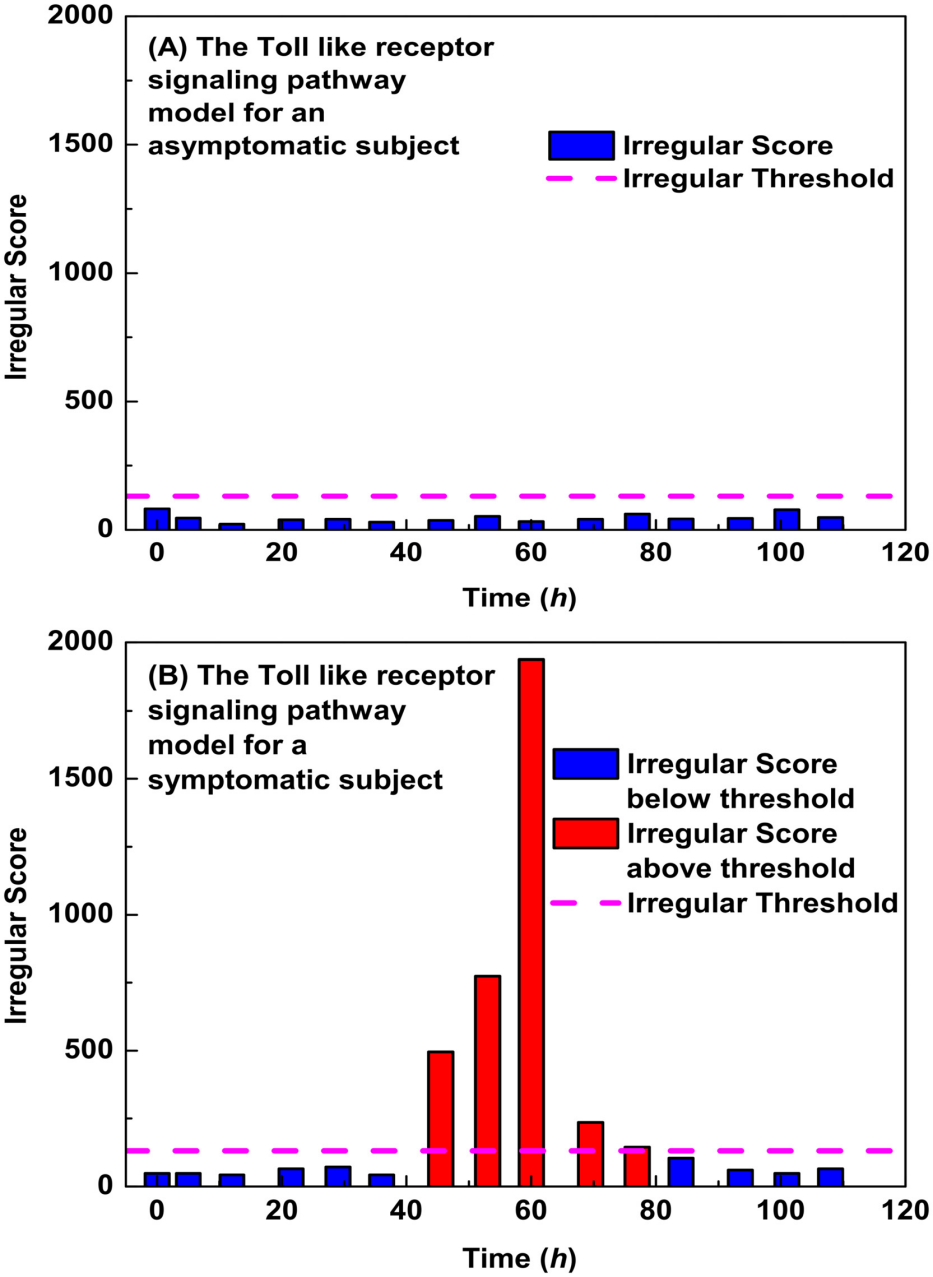
Evolution of the toll like receptor pathway residuals. The evolution of the toll like receptor pathway residuals for both an asymptomatic (A) and symptomatic (B) subject. When the residual level exceeds that critical threshold the pathway is deemed to be in alarm, indicating a response by the immune system to infection. The irregular score is the computed *χ*^2^ value of the residuals of the MSET model, and if it exceeds the threshold then the pathway is deemed to be in alarm.

**Fig 6:**
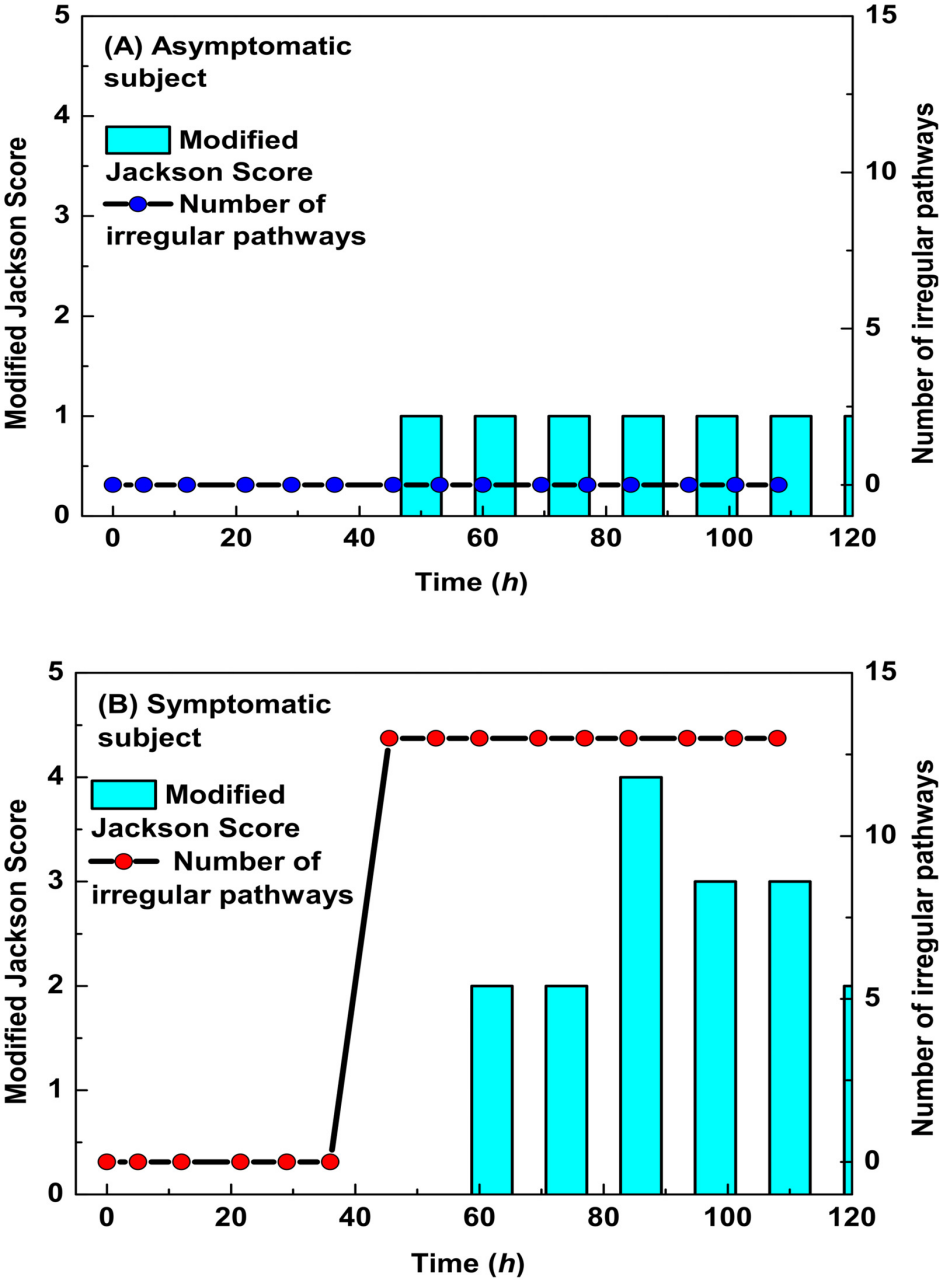
Temporal distribution of the respiratory virus pathway signature. The evolution of the most accurate pathways for predicting the development of symptoms for both an asymptomatic (A) and symptomatic (B) subject. The Jackson scores are a measure of how well the patient self reports his or her level of discomfort. The symptomatic subjects have all of our early warning pathways in alarm by 40 hours while these pathways behave nominally for the asymptomatic subjects.

It is interesting to further examine the explicit time-dependent behavior of the pathway models more closely. We applied the toll like receptor anomaly detection model to determine the prognosis for both an asymptomatic subject (A) and a symptomatic subject (B). The toll like receptor pathway did not detect an anomaly for the subject who remains asymptomatic while, in contrast, this pathway shows a clear anomaly for the symptomatic subject some forty hours after infection (Fig 5). We measured the response of the subset of most accurate diagnostic pathways, again for both an asymptomatic and symptomatic subject (Fig 6). Although the asymptomatic subject does feel somewhat unwell, as indicated by the Jackson Score, none of the most accurate pathways are in alarm. In contrast, these pathways all alarm in unison some 12 hours before the symptomatic subject begins to feel significantly ill. These results suggest that temporal pathway measurements can be exploited to monitor the host network response to respiratory virus infection.

### Anomaly detection of host pathways associated with endotoxin exposure

In order to detect host pathway anomalies associated with an endotoxin or bacterial infection we analyzed host response data sets that studied the acute inflammatory and immune response to understand the mechanism of LPS response over time between the endotoxin-treated and control groups [23, 34]. By analyzing changes in blood gene expression patterns in response to the inflammatory stimulus, the study reveals that the human blood leukocyte response to an acute systemic inflammation includes the transient dysregulation of leukocyte bioenergetics and modulation of its translational machinery. The dataset has 4 treated and 4 placebo subjects (see Table 2). We selected two of the four control subjects to create memory matrix. The remaining 6 subjects (2 placebo and 4 treated) were used as test data. Thus 6 MSET experiments in total were performed in this study. To obtain consistent results, the signaling pathways were selected as having consistent classification ability, and no misclassification for all 6 MSET experiments. There were numerous host pathways that ranked with high specificity and sensitivity using the MSET approach (Table 4). A total of 13 host pathways classified the cellular response to the endotoxin exposure in humans. The top 5 ranked pathways that distinguished endotoxin treated from healthy subjects with 100% accuracy were KEGG African Trypanosomiasis (1.00), KEGG Lysine Biosynthesis (1.00), BIOCARTA LYM (1.00), BIOCARTA SPPA (1.00), and BIOCARTA CDMAC (1.00). These pathways encompass cellular components responsible for pro-inflammatory responses, the recruitment of lymphocytes, blood platelet activation, and the proliferation of leukocytes, primarily macrophages. The activation of TLR4 has been shown to mediate the immune response to LPS and this host receptor induces a strong pro-inflammatory state by stimulating a classical M1 macrophage upon induction [35]. The top ranked endotoxin pathways identified in our MSET analysis represent host responses associated with an acute endotoxin exposure in humans mimicking a bacterial infection. Due to the rapid induction of host gene expression profiles in humans exposed to LPS, our MSET analysis detected anomalies all 13 endotoxin pathway classifiers in exposed subjects within 2.5 hours. These results show an immediate and robust host response to endotoxin exposure that is primarily driven by TLR4 mediated activation of immune cells in the blood. Interestingly, PECAM1 has been shown to regulate TLR4 signaling, preventing an excessive immune response and therefore possible damage [36]. It has also been shown in other studies that IL8, VCAM1, and ICAM1, which are other genes found in the LYM pathway, are induced by LPS. This too explains the anomalous expression of the LYM pathway induced by LPS [35]. IFNG and TNF, which are both found in the TID pathway, are shown to be induced by LPS [37–39]. This may explain the anomalous expression of this pathway. HSPA1A, also found in the TID pathway, seems to have a negative regulatory effect on pro-inflammatory cytokine production induced by LPS, suggesting it may play an important role in limiting an excessive immune response [40]. Finally, it has been suggested that JAK2 may be involved in the induction of LPS induced septic shock. This is because removal of JAK2 prevents septic shock from occurring [41]. In addition, the IL10 pathway plays a major role in the regulation of inflammatory cytokines in order to limit an excessive immune response. Expression of IL10 is shown to be induced by LPS through activation of TLR4 [42]. It is suggested that IL10 is able to specifically control production of the early effectors of endotoxic shock such as TNF [43]. It has been found that mice without the PML gene are resistant to LPS induced septic shock, suggesting that the PML gene plays a role in the response to LPS [44]. P53 may be important for the down regulation of response to LPS as the lack of P53 causes a higher production of pro-inflammatory cytokines to be produced [45]. CREBBP is known to be activated by LPS and can also be found in this pathway [46]. Finally, DAXX, a component of the PML pathway known for its role in apoptosis, is upregulated by LPS [47]. TLR4 is known to induce pro and anti-inflammatory cytokines in response to LPS [48]. CD13 in the SARS pathway has been shown to response to LPS and regulate TLR4 [49, 50]. NCL has also been shown to regulate the inflammation of alveolar macrophages induced by LPS [51]. Finally, GPT in the SARS pathway, is increased by LPS [52]. LPS regulates CD44 expression and stimulates endothelial cells to express SELE and SELP in MONOCYTE pathway [53–55]. IL22 in the IL22BP pathway, CBL in the CBL pathway, and IL3 in the IL3 pathway, are also induced by LPS [56–58].

**Table 4:** Signaling Pathways selected by MSET based on an exhaustive study. The performances are presented as mean (standard deviation). The pathways are sorted based on the average time to alarm.

Pathway (number of genes)	Time (*h*)
KEGG African Trypanosomiasis	2.00 (0.00)
KEGG Lysine Biosynthesis	2.00 (0.00)
BIOCARTA LYM	2.00 (0.00)
BIOCARTA SPPA	2.00 (0.00)
BIOCARTA CDMAC	2.00 (0.00)
BIOCARTA CBL	2.00 (0.00)
BIOCARTA P35ALZHEIMERS	2.00 (0.00)
BIOCARTA PML	2.00 (0.00)
BIOCARTA TEL	2.00 (0.00)
BIOCARTA SARS	2.00 (0.00)
KEGG Thiamine Metabolism	2.08 (0.41)
BIOCARTA IL22BP	2.08 (0.41)
BIOCARTA MONOCYTE	2.33 (0.76)
BIOCARTA IL3	2.42 (0.83)

### Host pathway signatures to distinguish acute viral versus bacterial infections in humans

**Fig 7:**
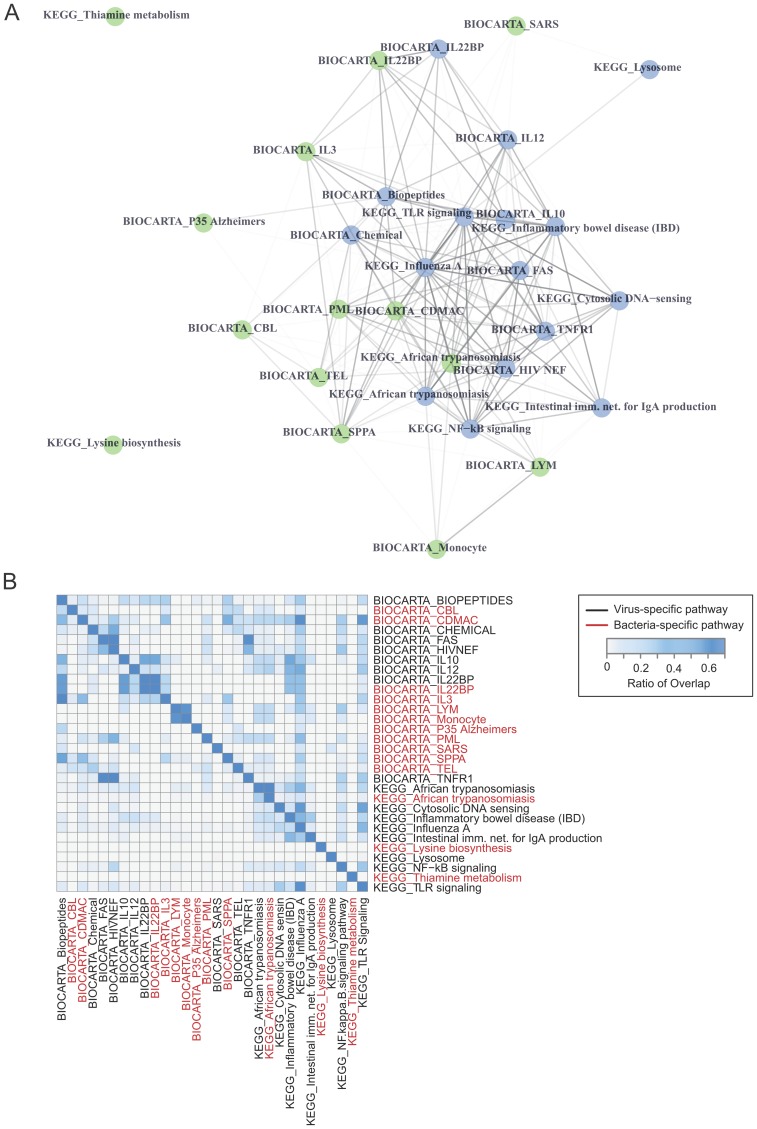
Evaluation of viral and bacterial signature redundancy. Weighted affiliation networks were generated to evaluate the gene redundancy across biological pathways that distinguish viral and bacterial signatures (A). Each node represents a pathway. Blue nodes denote viral-specific pathways and green nodes represent bacterial-specific pathways. Edges represent the connection between pathways based on the number of genes shared amongst each pathway. Edges are weighed on the basis of shared genes between pathways. The ratio of overlap between networks was evaluated and represented in the heat-map (B). Virus-specific pathways are denoted in black and bacterial-specific pathways are denoted in red in both the column and row labels.

We compared the top ranked pathway signatures generated from the 4 respiratory virus and endotoxin datasets to determine if our MSET pathway results could be used to differentiate between an acute bacterial and viral infection. The host pathway signatures defined by our analysis represent distinct cellular and immune signaling networks that show little overlap as far as biological function. Only two pathways, BIOCARTA IL-22BP and KEGG African Trypanosomiasis, were predicted in both the endotoxin (*n* = 13) and respiratory virus pathway signatures (*n* = 16). Affiliation networks demonstrated that the viral and bacterial pathways are connected and the majority of pathways share a subset of genes with at least one other pathway (Fig 7). Two bacterial pathways, Lysine biosynthesis and Thiamine metabolism, possessed unique gene sets that represent potential targets for differential diagnosis between viral and bacterial respiratory infections. Further analysis of the 526 respiratory virus vs. the 249 bacterial genes within these pathway signatures showed only 12.2% are commonly shared between the two pathogen signatures (Fig 7). These genes are directly involved in innate immune sensing (TLR, MYD88, JAK/STAT), and inflammation (IL-6, IFN, TNF, IL-10, IL-22) which are two common host signaling pathways activated by bacteria and viruses during acute respiratory infections. The vast majority of host genes found in the host pathway signatures were unique to the respiratory virus and endotoxin acute responses in humans (84% virus and 66% bacteria) providing a plethora of gene sets to evaluate for clinical differential diagnostic assays.

## Materials and Methods

### Data Overview

Here we describe the data sets used to illustrate the concept of early warning via anomaly detection of the immune response to infection. The first data set is generated by a numerical simulation of the immune system. We also consider three microarray data sets, four associated with respiratory viruses and one with endotoxin. We begin with a numerical simulation of the acute inflammatory response to pathogenic infection, i.e., sepsis, and generate illustrative data for this study [24, 59]. This model describes the generic response of the immune system to infection as captured by four bulk variables, i.e., pathogen level, neutrophils to capture inflammation, cytokines as a proxy for anti-inflammation and damage to tissue as a consequence of the immune response. This model based approach allows us to generate enough synthetic data to test and validate our early warning approach. In contrast to the other problems we explore, this study is data rich.

#### Transcriptomics Data

We examine five microarray data sets from the literature that were collected in association with disease challenges with human subjects as summarized in Table 2. We analyze four data sets associated with symptomatic respiratory viral infections in addition to an LPS experiment.

- *H*1*N*1: The *H*1*N*1 microarray experiment consists of 24 human subjects inoculated with influenza A (A/Brisbane/59/2007) [4]. There were 9 subjects that were excluded due to the indetermination. Thus the *H*1*N*1 dataset includes 9 subjects who developed symptoms and 6 subjects classified as asymptomatic.
- *H*3*N*2: The *H*3*N*2 microarray experiment consists of 17 human subjects inoculated with influenza A (A/Wisconsin/67/2005) [5]. Two subjects were excluded due to the indetermination. The *H*3*N*2 dataset has 9 symptomatic and 6 asymptomatic subjects.
- *HRV*: The *HRV* microarray experiment consists of 20 human subjects inoculated with Rhinovirus (HRV) serotype 39 [5]. The *HRV* dataset includes 10 subjects who developed symptoms and 10 subjects classified as asymptomatic.
- *RSV*: The *RSV* microarray experiment consists of 20 human subjects inoculated with respiratory syncytial virus (RSV) serotype A [5]. One subject had late symptoms and uninterpretable culture data and was excluded. Thus the *RSV* dataset includes 8 subjects who developed symptoms and 11 subjects classified as asymptomatic.

For both H1N1 and H3N2, the actual time points are -5, 0, 5, 12, 21.5, 29, 36, 45.5, 53, 60, 69.5, 77, 84, 93.5, 101, 108 hours. For HRV, peripheral blood was taken at baseline, then at 4 hour intervals for the first 24 hours, then 6 hour intervals for the next 24 hours, then 8 hour intervals for the next 24 hours, and then 24 hour intervals for the remaining 3 days of the study. 14 time points were found in the original data set without actual time provided. For RSV, peripheral blood was taken at baseline, then at 8 hour intervals for the initial 120 hours, and then 24 hours for the remaining 2 days of the study. 21 time points were found in the original data set without actual time provided. A summary of the number of data samples associated with data set is provided in Table 5. All subjects had peripheral blood samples taken prior to inoculation with virus (baseline), and at set intervals following inoculation. All four datasets are publicly available at: http://people.ee.duke.edu/~lcarin/reproduce.html. We investigate an endotoxin lipopolysaccharide (LPS) microarray experiment that included 8 human subjects [23, 34]. The gene expression levels were measured before infusion at 0 *h* and at 2, 4, 6, 9, and 24 *h* afterward. The *LPS* dataset consists of 4 subjects who were administered endotoxin and 4 who were administered a placebo. The *LPS* dataset is also publicly available at: http://www.gluegrant.org/pubsupport/Nature_1.

**Table 5:** Overview of the influenza datasets analysis. The baseline samples are less than validation samples because of missing baselines measures. For validation and test columns, the number of asymptomatic (asy) subjects and the number of symptomatic (sym) are also shown. And each subject has samples collected at set intervals after inoculation.

Selection	Baseline D	Validation *T*_0_ (asy/sym)	Test *T*_1_ (asy/sym)
*H*1*N*1, *H*3*N*2, *HRV*	47	50 (22/28)	19 (11/8)
*H*3*N*2, *HRV*, *RSV*	50	54 (27/27)	15 (6/9)
*HRV*, *RSV*, *H*1*N*1	50	54 (27/27)	15 (6/9)
*RSV*, *H*1*N*1, *H*3*N*2	45	49 (23/26)	20 (10/10)

#### Pathway Analysis Data

A collection of 511 pathways were used for the actual analysis of the gene expression data sets. These pathways map the multivariate interactions between genes associated with biological processes, such as metabolism, signal processing, and human diseases, based on biological knowledge. The pathways included in our analysis are comprised of

- 294 KEGG pathways (www.genome.jp/kegg),
- 217 BioCarta pathways (www.biocarta.com).

### Model Rational

Our hypothesis is that the immune system behaves *nominally* when the host is in a healthy state. We implement an anomaly detection framework that detects temporal changes in the evolution of a dynamic system. The assumption in the model building process is that there is no observation of anomalous behavior, only data associated with nominal (healthy) subjects is used for training a model function *f*(*x*(*t*)) where the pathway evolving in time may be viewed as a nonlinear curve observed over *T* time units

$$\begin{array}{c} x:\left[0,T\right]\to R^{n} \end{array}$$

where *n* is the number of genes in the pathway. One approach to anomaly detection is the construction of the mapping of the identity, i.e.,

$$\begin{array}{c} f(x(t))=x(t) \end{array}$$

for all points on the curve *x*(*t*) that are considered to be nominal. When this relationship fails to be true then we conclude that there is a novelty in the data and that the system for which the model was constructed has changed. At this point we refer to the model as being in *alarm*.

There are a number of approaches for constructing mappings of the identity for a given data set, see [60] for a general discussion. In this paper we restrict our attention to the Multivariate State Estimation Technique (MSET), a non-parametric statistical method that has been applied to detect anomalous system behavior in temporally evolving systems [16–21]. MSET uses a model of the system that applies under nominal operating conditions. As time evolves MSET is used to monitor the state variables of the system and to identify deviations from the nominal state as they occur, thus providing an early warning system for potential system failures. This approach has been effectively applied for monitoring large physical systems such as power plants [17] and NASA’s Space Shuttle. It is attractive for the current application given the absence of ad hoc parameters and the simplicity with which it can be trained.

The MSET model of a system is constructed from a historic sample of nominal data. In the current application this data will be the gene expression levels of healthy individuals. Since we are interested in understanding the immunological response we have organized the gene expression data by pathways. In this setting an MSET early warning system will be constructed for each pathway. Each of these MSET pathway models can now be used to monitor the temporally evolving system and identify departures from nominal state behavior.

Each model maps the given state of the system to a new state. If the system is operating in a nominal manner, the output of this mapping is effectively the same as the input to within some error tolerance. However, if the system’s operating characteristics have changed then the output of this mapping will no longer satisfy this property, i.e., the output of the MSET mapping will now deviate from the input by more than the allowed tolerance. It is standard practice with MSET to use the Sequential Probability Ratio Test (SPRT) to detect system alarms, i.e., critical deviations where the model is deemed to no longer apply to the system. In our application there are not enough data points (in time) to implement this approach so we implement a chi-square (*χ*^2^) test on the residuals as a means to identify alarm points. As the results indicate, we found this test to be very effective for detecting anomalies, but there is no theoretical basis to claim it is the optimal approach. Statistically significant outliers in the model residual are then used to indicate anomalous system behavior.

### Multivariate State Estimation Technique

**Fig 8:**
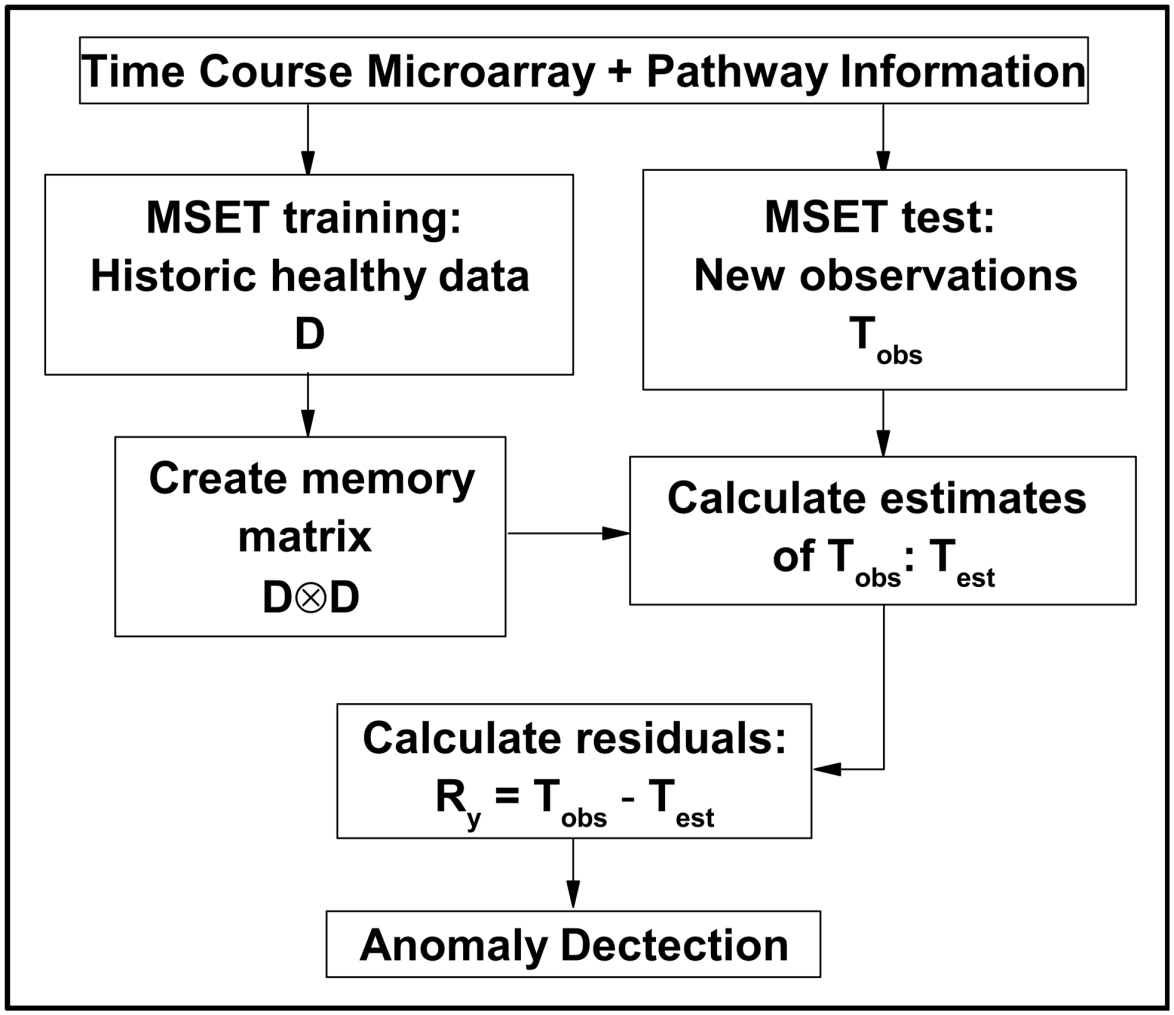
Schematic diagram. A schematic diagram of pathway-based anomaly detection for dynamic analysis using Multivariate State Estimation Technique [20]. The data is split into a training set which are used to build the model and a testing set which is used to validate it. When the model fails to describe the new data, the residuals become large and an anomaly is detected.

As shown in Fig 8, the training data *D*, also known as the memory matrix, consists of data collected while the state of system is deemed to be operating under nominal conditions. In this application the gene expression samples are collected from healthy individuals and organized by pathway, so there will be a memory matrix associated with the temporal evolution of each pathway state. Specifically, the data *D* associated with a given pathway is a *p* × *n* matrix that defined as

$$\begin{array}{c} D=[x\left(t_{1}\right),\cdots ,x\left(t_{i}\right),\cdots ,x\left(t_{M}\right)], \end{array}$$ (1)

where *x*(*t*_*i*_) is a *p*-dimensional vector measurement of a healthy state at time *t*_*i*_. The value *p* is the number of genes in the given pathway and will vary amongst pathways. The value *M* is the total number of healthy data states available for building the model. The expression levels of the *p* genes in a given pathway encode a component of the subject’s biological state. Thus the vector *x*(*t*_*i*_) consisting of the measurements of the *p* genes expression levels reflect the biological state of that pathway at time *t*_*i*_.

Each MSET pathway model is effectively a monitoring system that detects deviations in the gene expression patterns from the ideal healthy state. New measurements of gene expression levels, denoted by *y*_*obs*_, are mapped by MSET to model estimated states *y*_*est*_. As described below, if *y*_*obs*_ ≈ *y*_*est*_ then we conclude that the system is operating under nominal conditions.

The MSET mapping used to detect novelty is based on the construction of similarity operator in terms of the memory matrix *D* as [16–18]:

$$\begin{array}{c} y_{est}=D{\left(D^{T}⊗D\right)}^{-1}\left(D^{T}⊗y_{obs}\right), \end{array}$$ (2)

where the matrix *D*^*T*^ ⊗ *D* is called a *similarity matrix*.

The ⊗ notation is used here as a nonlinear operator that takes two matrices to produce a new matrix; it should not be confused with the more standard use of this notation for tensor product. It is defined component-wise as

$$\begin{array}{c} s\left(X^{\left(i\right)},Y^{\left(j\right)}\right)={\left(X^{T}⊗Y\right)}_{ij} \end{array}$$

where the function *s* encodes the similarity *X*^(*i*)^ and *Y*^(*j*)^, i.e., the *i*th and *j*the columns of *X* and *Y*, respectively. If we take

$$\begin{array}{c} s\left(X^{\left(i\right)},Y^{\left(j\right)}\right)={\left(X^{\left(i\right)}\right)}^{T}Y^{\left(j\right)} \end{array}$$

then the similarity amounts to the usual correlation and the MSET mapping is a projection onto the space spanned by the data. However, as proposed in [16], if one takes

$$\begin{array}{c} s\left(X^{\left(i\right)},Y^{\left(j\right)}\right)=1-\frac{||X^{\left(i\right)}-Y^{\left(j\right)}||}{||X^{\left(i\right)}||+||Y^{\left(j\right)}||} \end{array}$$

then the resulting matrix *X*^*T*^ ⊗ *Y* is a nonlinear measure of the similarity between *X* and *Y*. We note that while this measure has proven very effective, there are a variety of options that can be explored [16]. The vector *D*^*T*^ ⊗ *y*_*obs*_ measures the similarity of a given observation *y*_*obs*_ with each nominal sample *x* in the memory matrix *D*. It is easy to see that the MSET mapping acts as the identity mapping, i.e., maps a point to the identical point, on the observations that make up *D*. This is a consequence of the fact that

$$\begin{array}{c} D=D{\left(D^{T}⊗D\right)}^{-1}\left(D^{T}⊗D\right), \end{array}$$ (3)

which if we isolate a column of *D* means

$$\begin{array}{c} X^{\left(i\right)}=D{\left(D^{T}⊗D\right)}^{-1}\left(D^{T}⊗X^{\left(i\right)}\right). \end{array}$$ (4)

In other words, *X*^(*i*)^ gets mapped to itself.

For a newly observed state that shares similarities with the observations that make up the columns of *D*, the difference, or residual, between the estimate and observation is relatively small. Here the difference between a actual observation *y*_*obs*_ and its estimate *y*_*est*_, i.e., the residual *r*_*y*_, is defined as

$$\begin{array}{c} r_{y}=y_{obs}-y_{est}. \end{array}$$ (5)

The residual *r*_*y*_ is used as a signal to detect outliers for MSET. We assume that this vector of residuals is a sample of an independent and identically distributed (i.i.d.) random multivariate variable. For an observed state that is not similar to the columns of *D*, i.e., it contains some significant novelty, the residual is larger and the assumption that it is governed by an i.i.d. normal distribution no longer holds.

For all observations from the test data *T*_*obs*_, the estimates *T*_*est*_ are calculated using the memory matrix *D* by Eq 2. The test residuals *R*_*T*_ = *T*_*obs*_ − *T*_*est*_ are then obtained by Eq 5. The test residuals *R*_*T*_ represent the deviation of the system under test from its healthy operating condition *D* and are called actual residuals.

The outliers describe the abnormal data behavior, *i.e*., anomalous observations which are deviating from the normal data variability. Here, the standard outlier detection method, chi-square (*χ*^2^) test, is applied to detect the anomalous observations.

### Algorithm Implementation

For each data set we construct a model based on using a subset of *nominal* data, i.e., data that is assumed to be taken from subjects whose immune system is not responding to an infection. We partition each data set into subsets of size *m* and *n*, associated with either symptomatic and asymptomatic subjects, respectively. Further, the data are measured at the discrete time points labeled {*t*_1_, *t*_2_, ⋯, *t*_*M*_}. The data is divided into two groups, one for training the models of size *n*_*h*_, and one for testing the models, as shown in Fig 8. We note that only nominal data associated with healthy subjects sampled at baseline (*t* = −5) is used to train the models. The memory matrix *D*_*k*_ defined in Eq (1) associated with the *k*th pathway is constructed from the microarray data from the *n*_*h*_ healthy subjects selected at random and thus has size *p*_*k*_ × *n*_*h*_. Given there are *n* − *n*_*h*_ healthy test subjects and *m* symptomatic test subjects not in the training data set, the test data matrix for the *k*th pathway *T*_*k*_ has size *p*_*k*_ × (*m* + *n* − *n*_*h*_).

Using this notation the algorithm can now be summarized in five basic steps:

1. Pathway *k* consisting of *p*_*k*_ genes is assembled from the available microarray data for each of the *k* = 1,…,511 pathways under consideration. The data matrices *D*_*k*_ and *T*_*k*_ are created by these *p*_*k*_ gene expression levels that constitute pathway *p*_*k*_.
2. The test residuals *R*_*T*_*k*__ are calculated using *D*_*k*_ and *T*_*k*_ and the MSET mapping.
3. The mean *μ*_*k*_ and standard deviation *σ*_*k*_ for *R*_*T*_*k*__ are obtained to perform the *χ*^2^ test with *p*_*k*_ degrees of freedom.
4. For each subject in *T*_*k*_, the *χ*^2^ values are calculated over the time course {*t*_1_, *t*_2_, ⋯, *t*_*M*_}. These are used to perform anomaly detection. If an anomaly is detected we refer to the time of detection as the diagnosis time. A *P*-value of 0.005 is used as a cutoff between normal and anomalous observations. We refer to the the *χ*^2^ value as the *irregular score*.
5. The performances for the selected pathway, the classification accuracy and the average diagnosis time are computed.

### Generation of affiliation networks and overlap evaluation

Affiliation network and overlap analysis of genes represented within the viral and bacterial-specific biological pathways were generated using the R platform (v3.1.3). Graph adjacency evaluation and network visualization was done using the Bioconductor package ‘igraph’ (v0.7.1) [61]. Networks were visualized utilizing a Kamada-Kawai layout [62].

## Discussion

Advances in host gene expression technologies have provided a wealth of data on the host response to infectious diseases. The large data sets generated by microarray and RNA sequencing (RNA-Seq) requires analytical tools that utilize new algorithms that can exploit the information to derive biologically meaningful results. The current challenge is applying an analytical tool or mathematical model to identify the critical host genes or gene networks in an unbiased manner to develop targeted clinical diagnostic assays and host-derived therapeutics against pathogenic microorganisms. Host pathway analysis incorporates the functional linkage between gene sets to rapidly derived host gene signatures associated with an acute infection. We utilized MSET, a well-known approach in the anomaly detection field, to analyze acute respiratory virus and endotoxin gene expression datasets from exposed human subjects. Our dynamical systems based MSET analysis incorporated the temporal dynamics of the host pathways, i.e. the changes in host response to infection overtime, to identify early host pathway biomarkers associated with acute infection.

There are only a limited number of gene expression data sets publicly available that measure the human cellular response to acute infection. Generally they have low temporal resolution, e.g., samples every 12-24 hours, but detailed gene coverage that allows us to perform a pathway based modeling approach. Clinical data sets with temporal sampling also typically have a small number of subjects [4, 5, 23]. In contrast, the numerical simulations of virtual patients can generate finely sampled data in time for potentially millions of subjects, but in general capture only a limited number of variables. Thus the experimental and simulated datasets evaluated in this study each have aspects that provided different challenges to the proposed algorithm. This proof of concept demonstrates the applicability of mathematical algorithms, e.g., MSET, combined with tools from machine learning, to identify early changes in the host acute response to infection with high specificity and sensitivity.

Our results suggest that the anomaly detection framework can be used effectively to objectively identify key functional pathways or biomarkers that play a fundamental role in discriminating biological states such as symptomatic versus asymptomatic. The analysis provided a ranking of the most accurate diagnostic host pathways associated with respiratory virus infection or endotoxin exposure. From these we identified the pathways with superior prognostic properties in the sense that they alarm, i.e., display novelty first following infection with a virus or bacteria. The top ranked respiratory virus pathways across all 4 viral datasets (IL-22BP, IL-10, Fas, and intestinal IgA production) reveal an overall host signal generally associated with the intestinal mucosa and homeostasis of the gut epithelium. Respiratory viruses are known to cause gastrointestinal symptoms that are associated with direct infection of the intestinal epithelial cells and through modulation of the intestinal microbiome [63]. One study has linked early host immune activation in the gut to the efficacy of a live attenuated influenza vaccine administered intra-nasally in mice [64]. In addition, these pathways have been associated with immune cells (NK, T-cells) in the lung following infection with respiratory viruses [65]. Host gene expression analysis of whole blood samples primarily represents intracellular RNA from immune cell populations circulating in the bloodstream, so determining the tissue-specific source(s) of the immune cells that contribute to whole blood transcriptome profile is not feasible. The top functional pathways identified in the endotoxin gene expression data set (African Trypanosomiasis, Lysine Biosynthesis, LYM, SPPA) reveals host pathways involved in amino acid metabolism, epithelial barrier integrity, and immune cell proliferation/migration.

Early differential diagnosis between bacterial and viral respiratory infections would greatly enhance the treatment of acute respiratory infectious diseases. In this study, we defined pre-symptomatic or early warning pathway signatures for both data sets and found these pathogen-specific biomarkers could distinguish between a patient infected with a bacteria (12 pathways) vs. a virus (8 pathways) within 24 hours post exposure. The pre-symptomatic 8 pathway respiratory virus signature contains host-signaling networks involved with the innate antiviral sensing, inflammation, and mucosal integrity. These functional pathways are consistent with other studies predicting host biomarkers for respiratory diseases [66]. The pre-symptomatic 12 pathway endotoxin or pathogenic bacteria signature strongly correlates with macrophage/epithelial activation and pro-inflammatory responses (M1 response) primarily driven by LPS induced TL4 signaling [67, 68]. A more in-depth network analysis of the gene sets that define these early warning viral and bacterial pathways revealed 441 viral-specific and 183 endotoxin-specific genes that could be implemented into PCR-based diagnostic panel assays to distinguish between acute human infections of viral and bacterial etiologies. Furthermore, a subset of genes in endotoxin-specific host pathways, lysine biosynthesis and thymidine metabolism, that did not share any overlapping genes with virus-specific host pathways. These combined pathways represent 22 unique bacteria associated genes that are being investigated as prognostic host biomarkers for bacteria co-infections in respiratory virus positive patients admitted to the clinic.

Based on the results obtained here, we feel further evaluation of the anomaly detection approach for pathway analysis is warranted. We plan to further validate our results using other algorithms for discovering novelties in temporally evolving biomarkers, as well as supervised approaches for classification. By applying various anomaly detection approaches to human gene expression data sets with temporal sampling, we can define unique host gene classifiers that can distinguish between symptomatic (infected) and asymtomatic (uninfected) subjects. This will also permit additional elucidation of the complex processes of the host cellular response to infection, such as host signaling networks or functional pathways to diagnosis patients infected with different pathogenic etiologies (bacteria, viruses, fungi and parasites). As human data sets in response to acute infectious diseases becomes more readily accessible mathematical algorithms may be employed to identify host gene signatures that can predict infections in presymptomatic patients and distinguish between closely related viruses (SARS vs. MERS) and/or at the virus strain level (Influenza H1N1 vs. H3N2) that present similar disease manifestations. Rapid host-derived gene panels that represent pathogen-specific biomarkers could be developed to complement PCR-based assay panels that target pathogen genomes for more accurate clinical diagnostics of acute infectious diseases.
